# Tics of Trauma: Unique Case of Trauma-Associated Psychogenic Tics in an Adolescent Patient

**DOI:** 10.7759/cureus.17652

**Published:** 2021-09-01

**Authors:** Justine Ku, Cassidy Koo, Varsha Patel

**Affiliations:** 1 Psychiatry, University of California Riverside School of Medicine, Riverside, USA; 2 Psychiatry, Riverside University Health System (Corona Wellness Clinic), Riverside, USA

**Keywords:** pediatric psychiatry, psychogenic tics, sexual abuse trauma, case report, pediatrics, adolescent, family dynamics

## Abstract

Tic disorders are a common psychiatric diagnosis in adolescents. While often a primary disorder, there have been few reports of trauma-associated secondary psychogenic tics. In this case, we detail a 16-year-old girl with a history of trauma who initially presented with depression and trauma-related anxiety. During the course of treatment, she also developed verbal and motor tic-like symptoms that worsened with stress and court proceedings. We classify these new symptoms as psychogenic tics secondary to the trauma associated with sexual abuse. We explore the implication of psychiatric comorbidities, socio-legal stressors, and medication changes in the patient’s new-onset motor and vocal tics. This case points toward a need for consideration of this unique psychiatric manifestation of trauma-associated tics in adolescents with a history of sexual abuse and with otherwise normal neurological and physical examinations.

## Introduction

Tic disorders are often a primary diagnosis in adolescents. Less commonly, tics may develop secondary to medications or psychiatric comorbidities. There are only a few cases of psychogenic tics associated with trauma. This includes reported cases of pseudo-tics or psychogenic movements related to stressors, such as sexual abuse, in children previously diagnosed with Tourette Syndrome (TS). In one case, the child was later diagnosed with conversion disorder in addition to preexisting TS [1]. In another report, 19 teenagers developed sudden onset of tic-like movements of the upper extremities. Only two had prior diagnoses of a tic disorder; 18 were girls, and 10 had significant life stressors. All of their neurological exams were unremarkable. This case was later described as a mass psychogenic conversion disorder [2]. This report describes a unique case of psychogenic tics in an adolescent Hispanic female with post-traumatic stress disorder (PTSD), major depressive disorder (MDD), and a history of sexual abuse. She had no personal or family history of tics.

## Case presentation

A 16-year-old patient presented at our Child and Adolescent Psychiatry clinic for initial evaluation. She disclosed being molested by her biological father from seven years of age till when she was 12-years-old. She also reported symptoms of PTSD. The patient presented with symptoms of emotional dysregulation along with depression, agitation, and irritable mood. She was easily annoyed and distraught with her siblings. She demonstrated numbness, detachment, and avoidance of discussing the past. The patient was quickly startled and vigilant and reported intrusive thoughts of the bed and the house. The patient also had an auditory hallucination of a deep, scratchy male voice. 

**Figure 1:**
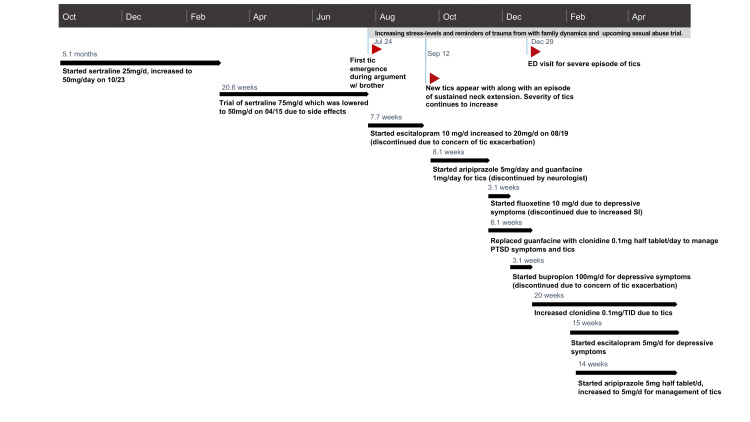
Significant events and medication treatment regimen Medication treatment regimen documented from initial evaluation to the present time (approximately 1.5 years). Significant events relating to the presentations of her tics are notated by the red arrows.

The patient had no other prior medical conditions. In addition to outpatient trauma-focused psychotherapy, various medications were used in the course of treatment (Figure 1).

First tic emergence

Initial pharmacologic management of her mood and PTSD symptoms consisted of sertraline (50-75 mg/day), which was effective for ten months. This sertraline dosing was adjusted and later discontinued due to side effects of daytime somnolence and lack of appetite, with a plan to switch to escitalopram 10 mg/day. Shortly after stopping sertraline and one day before starting escitalopram, the patient developed an abnormal neck and shoulder movement during a heated argument with the patient’s brother over the family’s separation. The patient also experienced stress related to her biological father’s decision to take the sexual abuse case to trial.

A complete neurological workup was performed: CT and EEG were unremarkable, laboratory tests were within normal limits, and evaluation by the neurologist showed no evidence of primary neurological disorder.

Increasing severity of tics

Due to her continued depression and increasing anxiety, escitalopram was increased to 20 mg/day. After one month, the patient developed a new tic in which her head jerked backward. She also experienced episodes of raising the right shoulder and lateral flexion of the neck to make an impact with her shoulder. These tics were frequent (occurring 10-20 times per day) and severe (developing bruises on her shoulder and cramps in her neck). The patient and family decided to stop escitalopram 20 mg/day because it was increasing the severity of the patient’s motor tics. 

After discontinuation of escitalopram, the patient had decreased frequency and intensity of movements. During this time, the patient continued to be stressed over court deadlines to testify against her father regarding the sexual abuse charges. She experienced a new episode of sustained neck extension for 45 minutes. The patient’s neurologist started her on aripiprazole 5 mg/day (taken for two months) and guanfacine 1 mg/day with improvement in her tics. 

Mild improvement in tics

After two months, the patient continued to have mild tics approximately 10 times per day, consisting of abrupt neck movement and occasional hand movements in which she dropped objects. She also experienced nightmares of her biological father standing next to her and of her parents getting remarried. As the patient continued to have a depressed mood, she was started on a regimen of fluoxetine 10 mg/day. Guanfacine was also replaced with clonidine 0.1 mg/day to better manage the patient’s PTSD symptoms and tics. Subsequently, the patient reported improved mood and decreased shoulder tic frequency, with no change in the neck tics. Fluoxetine was later discontinued due to concern for increased suicidal ideation. To treat her depression and PTSD symptoms, such as flashbacks, she began taking bupropion SR 100mg/day. 

Emergency department visit for tic episode

After one month on bupropion, the patient had a severe episode of tics and was taken to the emergency department. The patient experienced both motor tics and a new verbal tic of saying a profanity word and “up". This tic seemed to be triggered by hearing hospital staff around her say the word “up” while describing her ECG. Due to this unprecedented incident, bupropion SR was discontinued due to fear that it was exacerbating the patient’s tic disorder.

In the next month’s follow-up appointment, the patient reported no improvement in her neck motor tics. She also had increasing anxiety about her upcoming meeting with the district attorney to discuss testifying against her father in court. During this clinic visit, the patient reported further details of the sexual abuse. The patient recalled incidents of abuse when she felt smothered and had difficulty breathing, causing her to jerk her head away from the perpetrator in response. Her mood was anxious, depressed, detached, numb, and vigilant. It was decided to cautiously reinstate escitalopram at 5 mg/day. On reinstating escitalopram, the patient felt a slight decrease in irritability and interpersonal sensitivity but continued to have motor tics. She also developed a new tic of hitting her head with the left or right hand. This was so severe that she could not hold her fork during meals. In one incident, the patient splashed spaghetti on the wall when her hands jerked backward. At this time, we restarted her on aripiprazole 5 mg half tablet/day to control tic symptoms.

Ongoing tics

The patient continues to be anxious about testifying in court and seeing her biological father at the trial. During this time, she continues to develop new tics, including her arm hitting the countertop and her thigh. During her most recent appointment, she also demonstrated suggestibility of the vocal tic, saying “up” after that word was repeatedly mentioned. The patient’s tics are better during the daytime and worse at night. They also increase during periods of stress, cold weather, excitement, or interaction with her siblings. However, both the patient’s tics and mood symptoms have shown improvement on a current regimen of escitalopram 5 mg/day, clonidine 0.1 mg three times daily, and aripiprazole 2.5 mg half tablet/day in addition to outpatient trauma-focused psychotherapy. 

## Discussion

We postulate that this patient’s tic-like movements were secondary to the trauma associated with her history of sexual abuse, with the possibility that medication treatment predisposed the patient to the development of these tics. The described causes of tic disorders have ranged vastly from dysfunctional pathways between the cerebral cortex and basal ganglia to the involvement of sensory limbic and executive corticostriatal loops. However, a widespread, supported consensus suggests that the neuronal disinhibition associated with the sensory and motor component of a tic is due to a change in the signaling of dopamine and histamine neurotransmitters [3,4]. On reviewing the patient’s medications, we investigated sertraline, escitalopram, and bupropion as potential medication causes to the patient’s condition. Each of these medications has reported secondary action in modulating dopamine levels or systems [5-7]. In one study of sertraline, fluvoxamine, and paroxetine in rat models, sertraline was found to increase dopamine concentrations in the nucleus accumbens and striatum [6]. There are also few reports of sertraline exacerbating tics in humans [8-9]. Most case reports of sertraline-induced tics occurred within one month of initiation with sertraline and resolved after discontinuation of sertraline [8-9].

In contrast, our patient did not develop tics while on continuous sertraline monotherapy for ten months and continued to develop tics of increasing severity even after discontinuation of sertraline. We found only one case in the literature documenting the persistence of tics after discontinuation of sertraline [10]. While it is unlikely that sertraline was the sole proponent of this patient’s tics, we cannot rule out the possibility that sertraline may have played a role in modulating dopamine pathways and levels leading to a predisposition for tic emergence. 

Escitalopram was also considered as a factor in the patient’s new-onset tic disorder. We found one reported case of a woman on escitalopram who developed facial tics, which resolved two weeks after discontinuation [9]. In contrast, our patient said that her initial tic episode occurred one day before starting escitalopram and did not improve with discontinuation. She also experienced improved mood and decreased severity of tics on re-initiating escitalopram, making this medication a less likely suspect in the exacerbation of her tics. Concerning bupropion, we found reports of a 19-year-old man who developed shoulder and verbal tics after initiating bupropion [11], as well as a series of four patients with attention deficit hyperactivity disorder (ADHD) and TS who experienced tic exacerbation when treated with bupropion [12]. In all cases, discontinuation relieved symptoms, while in contrast, the new tics persisted in our patient even after discontinuing bupropion. In addition, our patient’s initial tic emergence began months before the initiation of bupropion. As a result, bupropion is also a less likely proponent of tic emergence in this patient. 

While it is possible that the patient’s 10-month duration of sertraline monotherapy led to tic predisposition via increased baseline levels of dopamine, this case emphasizes the critical role of psychogenic stress in contributing to the development of a new-onset tic disorder. We propose that in the setting of unremarkable physical and neurological examinations and medication predisposition, the final manifestation of tics was likely directly induced by psychogenic stress related to complex family dynamics and reminders of the trauma. As a result of the patient disclosing sexual abuse, her siblings blamed her for their family’s separation. She also had to leave her home and adjust to living with a new legal guardian. In addition to these environmental stressors, the patient was preparing to testify in court and immersed in reminders of the sexual abuse. Her recollection of details about the sexual abuse paralleled the timing and presentation of her pervading neck, shoulder, and arm tics. For example, the tics increased in severity during the evenings, which was also the reported time most of the sexual abuse occurred. The patient also reported incidents of abuse when she felt smothered and had difficulty breathing. In those cases, the patient recalled jerking her head away from the perpetrator in response. Her tics later reflected this same motion of jerking her head backward. 

Lastly, this case posed unique challenges during the COVID-19 pandemic. The patient’s prognosis may have been affected by increased isolation, lack of structure in virtual high school, and lack of group therapy during the pandemic. This social isolation limited her reprieve from her psychological traumas. This experience of trauma and the loneliness of being a victim can lead to a cycle of revictimization and alienation. We propose that the patient’s ongoing life stressors and trauma reminders manifested as these psychogenic tics of trauma.

## Conclusions

Both medical and psychiatric investigations are essential in the course of new-onset tics in adolescents. As in our patient, a full neurological workup should be completed, and a thorough investigation into the course of medication timing and potential side effects. In cases of new-onset tics in adolescents, it is paramount to screen for the entire medication history and psychogenic causes as inciting factors. This consideration can lead to appropriate, targeted therapy, especially in patients with a history of sexual abuse.
